# Tailoring treatment to the individual in type 2 diabetes practical guidance from the *Global Partnership for Effective Diabetes Management*

**DOI:** 10.1111/j.1742-1241.2009.02227.x

**Published:** 2010-02

**Authors:** S Del Prato, J LaSalle, S Matthaei, C J Bailey

**Affiliations:** 1 University of PisaPisa, Italy; 2 Medical Arts Research Collaborative, Excelsior SpringsMO, USA; 3 Diabetes-Center QuakenbrückQuakenbrück, Germany; 4 Aston UniversityBirmingham, UK

## Abstract

Good glycaemic control continues to be the most effective therapeutic manoeuvre to reduce the risk of development and/or progression of microvascular disease, and therefore remains the cornerstone of diabetes management despite recent scepticism about tight glucose control strategies. The impact on macrovascular complications is still a matter of debate, and so glycaemic control strategies should be placed in the context of multifactorial intervention to address all cardiovascular risk factors. Approaches to achieve glycaemic targets should always ensure patient safety, and results from recent landmark outcome studies support the need for appropriate individualisation of glycaemic targets and of the means to achieve these targets, with the ultimate aim to optimise outcomes and minimise adverse events, such as hypoglycaemia and marked weight gain. The primary goal of the *Global Partnership for Effective Diabetes Management* is the provision of practical guidance to improve patient outcomes and, in this article, we aim to support healthcare professionals in appropriately tailoring type 2 diabetes treatment to the individual. Patient groups requiring special consideration are identified, including newly diagnosed individuals with type 2 diabetes but no complications, individuals with a history of inadequate glycaemic control, those with a history of cardiovascular disease, children and individuals at risk of hypoglycaemia. Practical guidance specific to each group is provided.

**What's known**

- While good glycaemic control remains the cornerstone of diabetes management, strategies to achieve glycaemic targets should always ensure patient safety.
- Results from recent landmark outcome studies support the need for early, appropriate and individualised intervention.

**What's new**

- This article identifies patient groups requiring special consideration and provides practical guidance specific to each group.

## Introduction

Good glycaemic control remains the cornerstone of diabetes management despite recent scepticism about tight glucose control strategies and concerns regarding safety of highly intensive treatment in the ACCORD study (1). Recent publications, including our ‘Updated Recommendations from the *Global Partnership for Effective Diabetes Management*’ (2), continue to emphasise the essential role of good glycaemic control in reducing the risk of diabetes complications, although attempts to achieve glycaemic targets should always ensure patient safety. Some individuals may be unsuited to a particularly aggressive glucose-lowering regimen, for example, as illustrated in ACCORD, where all-cause mortality, risk of hypoglycaemia and weight gain were increased in patients exposed to this approach. Indeed, the treatment strategy adopted in this trial cannot be used as a benchmark for achieving optimal glycaemic control.

Ensuring good glycaemic control remains the most effective therapeutic manoeuvre to reduce the risk of development and/or progression of microvascular disease. In contrast, the impact on macrovascular complications is still a matter of debate. In the most recent outcome studies, ACCORD, ADVANCE and VADT (1,3,4), lowering HbA_1c_ below 7% was not associated with any significant reduction in cardiovascular (CV) mortality. However, these results require further critical appraisal. For example, the overall CV mortality was much lower than the one initially used to calculate the sample power of these studies. Such an unexpectedly low mortality rate most likely reflects the substantial changes in type 2 diabetes management over the past 10 years that have incorporated multifactorial treatment strategies. These include extensive use of statins, newer and more effective agents for hypertension, increased use of aspirin and smoking cessation programmes. With such a comprehensive approach to patient care, it is more difficult to demonstrate a significant risk reduction that can be assigned to glucose lowering alone.

Another possible reason for these observations may be that the study duration of ACCORD, ADVANCE and VADT was too short to show a significant effect of intensive glucose control on macrovascular complications. In contrast, in the 10-year UKPDS follow-up, relative risk reductions for myocardial infarction (MI) and all-cause mortality were significantly lower in patients who initially received intensive treatment compared with those in the conventional treatment arm. Moreover, the initial benefit in terms of microvascular complications observed at the end of the intervention trial remained unaltered at follow-up. There was, however, quite a difference between the patients enrolled in the UKPDS compared with those in the ACCORD, ADVANCE and VADT trials: newly diagnosed patients with no prior CV events in the former, subjects with long-standing disease and a high prevalence of microvascular and macrovascular complications in the latter.

When all the large, long-term, prospective randomised controlled clinical trials (UKPDS, PROactive, ADVANCE, VADT and ACCORD) are included in a meta-analysis, blood glucose lowering appears to be associated with reduction of incident CV events (5). Overall, a 0.9% reduction in HbA_1c_ with intensive therapy was associated with significant reductions of 17% in non-fatal MI [odds ratio (OR): 0.83, 95% confidence interval (CI): 0.75–0.93] and 15% in coronary heart disease (OR: 0.85, 95% CI: 0.77–0.93) vs. conventional therapy (5). In a meta-regression analysis, higher body mass index (BMI), duration of diabetes and incidence of severe hypoglycaemia were associated with greater risk of CV death in intensive treatment groups (6).

Altogether, these results support the need for appropriate individualisation of glycaemic targets and of the means to achieve these targets. Several factors can be taken into consideration when tailoring treatment including duration of diabetes, stage of disease, life expectancy, risk of hypoglycaemia and risk factors for CV disease (CVD). It should also be noted that even apparently acceptable levels of HbA_1c_ can disguise wide daily fluctuations in plasma glucose that require control. Although these considerations may sound relatively straightforward, confusion exists in the healthcare community regarding how this information can be translated into clinical practice.

The primary goal of the *Global Partnership for Effective Diabetes Management* is the provision of practical guidance to improve patient outcomes in diabetes. Our recommendations (2) have been updated after publication of the most recent outcome trials to incorporate a range for target HbA_1c_ (6.5–7%) to provide flexibility to suit different patient populations (2). In this article, we aim to build on this by providing more explicit advice to support healthcare professionals in appropriately tailoring type 2 diabetes treatment. This includes: (i) identification of patient groups requiring special consideration, including newly diagnosed individuals with type 2 diabetes but no complications (overweight or obese adults, lean adults and children), individuals with a history of inadequate glycaemic control (no complications or history of CVD) and individuals at risk of hypoglycaemia; and (ii) provision of practical guidance specific to each group.

Note that, given the existence of considerable national and regional differences in the availability of therapeutic options, recommendations concerning the use of particular antidiabetic agents have been avoided. Other regional differences, e.g. variations in phenotype and genotype, should also be acknowledged. In addition, we have not included individuals with good glycaemic control as a separate population. For these patients, the guidance is to maintain the same regimen to keep patients at target and to react quickly if HbA_1c_ starts to rise, e.g. by timely introduction of combination therapy or insulin as appropriate.

## Newly diagnosed individuals with type 2 diabetes, but no complications

### Overweight or obese adults

*Definition of patient type*: HbA_1c_ > 6.5%, BMI > 25 kg/m^2^, typically > 30 years of age, diagnosis before emergence of complications, mild symptoms or asymptomatic, no associated comorbidities, e.g. hypertension, dyslipidaemia.

As reviewed in previous *Global Partnership* publications (2,7–9), early and effective intervention to improve glycaemic control is likely to confer the greatest benefits. Achieving such a goal, however, requires a proactive approach including definition of target HbA_1c_ and prompt reaction at the first sign of any deviation from the target. This is in contrast with the traditional stepwise approach to type 2 diabetes management, which often leads to significant delays in both achieving and maintaining glycaemic goals and may result in long periods of hyperglycaemia. This approach is unacceptable given the evidence that even short periods of hyperglycaemia increase the risk of microvascular and macrovascular complications (7).

The benefits of early intervention are illustrated by results from the follow-up to large-scale outcome studies in both type 1 and type 2 diabetes, which have led to the proposal of a metabolic memory or metabolic legacy effect (10–14). The available evidence suggests that early, strict glycaemic control may confer protection against, or delay, the serious long-term complications of the condition. For example, during 8-year follow-up of the DCCT/EDIC study in type 1 diabetes, earlier improvements in glycaemic control resulted in continued microvascular benefits as well as the emergence of macrovascular risk reduction over time (11–14). Similarly, during 10 years’ follow-up of type 2 diabetes patients in the UKPDS, a continued reduction in microvascular risk as well as emergent reductions in the risk of MI and death from any cause was observed with intensive vs. conventional therapy (10). This was seen despite an early convergence of HbA_1c_ levels between the groups. Potential mechanisms to explain the molecular basis for metabolic memory include increased formation of cellular reactive species and advanced glycation end products in response to chronic hyperglycaemia, resulting in the activation of pathways involved in the pathogenesis of diabetes-related complications (15,16).

Intervention later in the course of the disease provides less opportunity to influence the development and/or progression of complications in patients with long-standing diabetes. This is demonstrated by data from ACCORD, in which subgroup analyses of the intensively controlled group indicated a significant reduction in the primary CV end-point in individuals with HbA_1c_≤ 8% at baseline or those who had not had a CV event before randomisation (1). Further evidence comes from the meta-regression analysis of the outcome trials clearly indicating that the earlier the intensive treatment, the larger the risk reduction of CVD (6). The same analysis suggests that the magnitude of the risk reduction decreases in individuals with longer disease duration with an increase in CVD risk in those with long-standing diabetes (17).

**Figure 1:**
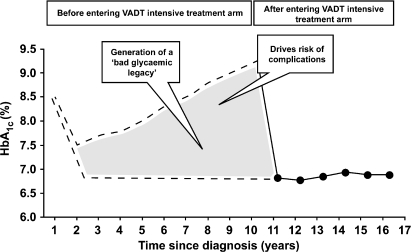
Estimated glycaemic legacy of patients recruited in VADT. Reproduced with kind permission of Springer Science and Business Media from Del Prato S. Megatrials in type 2 diabetes. From excitement to frustration? *Diabetologia* 2009; **52:** 1219-26.

All this can be readily appreciated by considering Figure 1, which illustrates the drawbacks of late intervention by hypothetically reconstructing the natural history of patients recruited into the VADT. Patients with poor glycaemic control (HbA_1c_ 9.4%) and long-standing diabetes (11.4 years) were recruited in the study. The solid line represents changes in HbA_1c_ over time in the VADT (18). After entry into the VADT intensive treatment arm, HbA_1c_ decreased rapidly and was subsequently maintained close to target levels. However, although diabetes progression up to this point remains unknown, it can be tentatively reconstructed based on data from the UKPDS as a case study. Thus, the upper broken line in Figure 1 represents the time course of HbA_1c_ estimated on the basis of the average glucose profile observed in the UKPDS (19), while the lower broken line represents the ideal time course of glycaemic control. The difference between the ideal and the actual time course of glycaemic control represents a time period that must have had a negative effect on subsequent tight glycaemic control. Clearly, reducing hyperglycaemia as early as possible is likely to reduce risk of complications.

#### Practical guidance for newly diagnosed overweight or obese adults

Current recommendations for this and other groups of patients are summarised in Table 1. The points of particular importance for this type of patient follow.

**Table 1:** Individualising the ‘10 steps to get more type 2 diabetes patients to goal’ according to patient type

	**Patient type**					
Recommendation*	**Newly diagnosed, no complications, overweight/obese adults**	**Newly diagnosed, no complications, lean adults**	**Newly diagnosed, no complications, children**	**History of inadequate glycaemic control, no complications**	**History of inadequate glycaemic control and CVD**	**Individuals at risk of hypoglycaemia**
Aim for good glycaemic control, e.g. HbA_1c_ 6.5–7%† when safe and appropriate	Aim for HbA_1c_ as close to normal as can safely be achieved without hypoglycaemia or marked weight gain	Aim for HbA_1c_ as close to normal as can safely be achieved without hypoglycaemia	Aim for HbA_1c_ as close to normal as can safely be achieved without hypoglycaemia	Aim for near–normal HbA_1c_ but more gradual reduction in HbA_1c_	Aim for less stringent HbA_1c_ targets and more gradual reduction in HbA_1c_, particular care to avoid hypoglycaemia	Aim for less stringent HbA_1c_ targets and more gradual reduction in HbA_1c_, particular care to avoid hypoglycaemia
Monitor HbA_1c_ every 3 months in addition to appropriate glucose self-monitoring	✓	✓	✓	✓	✓	Emphasise importance of self-monitoring of glucose
Appropriately manage all cardiovascular risk factors	✓	Lower risk of CVD, educate on lifestyle to avoid weight gain and associated CVD risk	✓	✓	Intensify cardiovascular risk management	✓
Refer all newly diagnosed patients to a unit specialising in diabetes care where possible	✓	✓	✓	–	–	–
Address the underlying pathophysiology of diabetes, including the treatment of β-cell dysfunction and insulin resistance	✓	Include agents that stimulate the β-cell where possible	✓	✓	✓	✓
Treat to achieve appropriate target HbA_1c_ within 6 months of diagnosis	✓	✓	✓	✓	✓	✓
After 3 months, if patients are not at target HbA_1c_, consider combination therapy	✓	Earlier introduction of combination therapy may be required because of increased likelihood of β-cell dysfunction	✓	✓	✓	✓
Consider initiating combination therapy or insulin for patients with HbA_1c_ > 9%	✓	✓	✓	✓	✓	✓
Use combinations of antihyperglycaemic agents with complementary mechanisms of action	✓	✓	✓	✓	✓	✓
Implement a multidisciplinary team approach that encourages patient self-management, education and self-care, with shared responsibilities to achieve goals	✓	✓	Include patient and family at centre of team and counsel other family members to prevent onset of diabetes	✓	✓	✓
Other	–	Recognise increased risk of LADA	Extra vigilance regarding long-term safety	Reassess reasons for inadequate glycaemic control and implement structured educational programmes	Extra vigilance regarding contraindications and drug interactions	Educate to increase awareness and responsiveness to hypoglycaemia, particularly in vulnerable patients such as elderly people

*Taken from the *Global Partnership’s* recommendations, updated in 2009 (2). †Or fasting/preprandial plasma glucose 110–130 mg/dl (6.0–7.2 mmol/l) where assessment of HbA_1c_ is not possible. CVD, cardiovascular disease; LADA, latent autoimmune diabetes in adults.

- Newly diagnosed type 2 diabetes patients with no evidence of complications should aim for the bottom of the HbA_1c_ target range (6.5–7%), i.e. HbA_1c_ as close to normal as can safely be achieved without causing hypoglycaemia or marked weight gain. HbA_1c_ should be monitored every 3 months, and initiating combination therapy should be considered if target HbA_1c_ is not reached within 3 months.
- Note the need to aim for normal HbA_1c_ wherever appropriate, even in patients with modest hyperglycaemia (HbA_1c_ < 7.5%).
- In patients with mild to moderate hyperglycaemia (HbA_1c_ < 7.5%), consider agents that are not associated with an increased risk of hypoglycaemia and that address the underlying pathophysiology of diabetes, including the treatment of β-cell dysfunction and insulin resistance.
- In patients with HbA_1c_ >9%, consider initiating combination therapy or insulin.
- For overweight or obese patients, as for all subjects with type 2 diabetes, diet and exercise should be continually reiterated.
- As overweight and obese patients are at increased risk of CVD, particular attention should be given to manage all CV risk factors.

### Lean adults

*Definition of patient type*: HbA_1c_ > 6.5%, BMI < 25 kg/m^2^, typically > 30 years of age, diagnosis before emergence of complications, mild symptoms or asymptomatic, no associated comorbidities, e.g. hypertension, dyslipidaemia.

Although the majority of individuals with type 2 diabetes are overweight or obese, a substantial number are considered lean by traditional standards, particularly in Asian countries (20). Notably, the proportion of type 2 diabetes cases attributable to obesity varies greatly across the world, from almost 90% in North America to < 40% in Southeast Asia (20). There is some evidence that the percentage of body fat for a given BMI is higher amongst certain Asian populations, which has led to the introduction of lower waist circumference and BMI thresholds for defining overweight/obese patients in some countries, e.g. Japan and China (20).

A major consideration in lean patients is the degree of β-cell dysfunction, and this may affect the choice of agent. β-cell dysfunction may be more marked in lean patients compared with overweight/obese individuals, and this is particularly true in some non-Western populations. For example, in a study of Korean type 2 diabetes patients with BMI < 25 kg/m^2^, only 24% was found to be insulin resistant (20). However, pathophysiological heterogeneity is also often increased in lean patients, which presents a particular therapeutic challenge. Given this heterogeneity and the practical difficulties of determining the exact degree of insulin resistance and insulin deficiency, a rational approach may be to ensure that both defects are addressed in the therapeutic regimen (20). Also, as ethnic and phenotypic variations across regions of the world appear to provide different contributions to the pathophysiology of the condition, it is important that this diversity is reflected in the treatment approach.

Lean patients may have a different profile in terms of susceptibility to macrovascular disease compared with overweight or obese individuals, with fewer risk factors for CVD and a lower risk of macrovascular vs. microvascular complications (20,21). Hence, the primary focus for lean patients is likely to be glycaemic control, while in overweight or obese patients multifactorial intervention to address CV risk factors such as dyslipidaemia and hypertension as well as hyperglycaemia may be more urgent (20).

#### Practical guidance for newly diagnosed lean adults

- Newly diagnosed type 2 diabetes patients with no evidence of complications should aim for the lower end of the HbA_1c_ target range (6.5–7%), i.e. HbA_1c_ as close to normal as can safely be achieved without causing hypoglycaemia. HbA_1c_ should be monitored every 3 months and initiating combination therapy should be considered if target HbA_1c_ is not reached within 3 months.
- Note the need to aim for normal HbA_1c_ wherever appropriate, even in patients with modest hyperglycaemia (HbA_1c_ < 7.5%).
- Ideally, use agents that are not associated with an increased risk of hypoglycaemia.
- In patients with HbA_1c_ >9%, consider initiating combination therapy or insulin.
- Given the increased likelihood of β-cell dysfunction in newly diagnosed lean type 2 diabetes patients, there may be a particular need for earlier therapy (including early use of combination therapy) in this group, including agents that support β-cell function wherever appropriate.
- Although this group has a lower risk of CVD compared with overweight or obese patients, individuals should still be educated on the importance of maintaining a healthy lifestyle to prevent weight gain, with its associated risk of CV complications.
- Be aware that latent autoimmune diabetes in adults (LADA), which constitutes around 10% of all diagnosed type 2 diabetes patients (20), may be more prevalent in lean patients. Therefore, it may be advisable wherever possible to test for islet autoantibodies, such as antibodies to glutamic acid decarboxylase, if LADA is suspected.

### Children

Although type 1 diabetes is traditionally more common in children and adolescents, the burden of type 2 diabetes is increasing in young people with the condition, with as many as 8–45% of new-onset paediatric diabetes cases in the USA attributed to type 2 diabetes (22). The prevalence of type 2 diabetes is also escalating in other regions, including Japan and the UK (23,24). With this increasing prevalence comes a pressing need to prevent or delay the development of serious diabetes complications. In particular, onset of the disease at such an early age signals the possibility of a significant glycaemic legacy if diabetes is uncontrolled over a long time period. Note that it is becoming increasingly difficult to distinguish between type 1 and type 2 diabetes in children given the significant overlap between presenting conditions. This has created a dilemma in terms of selecting the most appropriate management strategy. Moreover, as this phenomenon becomes increasingly common, new treatment strategies will need to be explored.

Involvement of a multidisciplinary approach to patient care, with the affected individual and their family taking a central role, is particularly important for children with type 2 diabetes. Inclusion of family members and friends can be particularly beneficial for individuals in terms of improving both diabetes-related knowledge and glycaemic control (25). Furthermore, many children with type 2 diabetes are overweight at diagnosis and most are in families with other members who are at high risk of diabetes, so lifestyle counselling and modification can involve the whole family (23). However, not all cases are associated with overweight or obesity, notably those in Asian and Oriental populations. As with all individuals with type 2 diabetes, access to structured educational programmes is essential (25).

#### Practical guidance for newly diagnosed children

- Extra vigilance regarding long-term safety is paramount in children with type 2 diabetes.
- Given that individuals are facing decades of the disease, aim for HbA_1c_ targets in the normal range without causing hypoglycaemia.
- Implement a multidisciplinary team approach to diabetes care, with both the patient and their family at the centre of the team. Involve the whole peer group, including parents, carers and family, in education regarding the importance of lifestyle and counsel other family members, e.g. siblings, to prevent development of diabetes.

## Individuals with a history of inadequate glycaemic control (lean or obese)

### No complications

*Definition of patient type*: Likely to be older than newly diagnosed individuals with no complications and with a longer duration of diabetes, e.g. inadequate glycaemic control (HbA_1c_ > 7.5%) for ≥ 1 year, no associated comorbidities, e.g. hypertension, dyslipidaemia.

Reducing microvascular and macrovascular complications represents a particular challenge in individuals with more advanced disease and therefore a high glycaemic burden. Before making treatment decisions, it is necessary to consider the reasons for an individual being inadequately controlled. Explanations may include poor adherence, lack of awareness of the benefits of good glycaemic control, conservative or delayed use of combination therapy or insulin by physicians and lower levels of β-cell function in these patients (7).

If patients have a history of inadequate glycaemic control this will confer a bad glycaemic legacy, as described above. In those patients who have no complications to date, it is important to lower HbA_1c_ levels to near normal to reduce the risk of complications, while balancing the benefits of good glycaemic control with patient safety.

#### Practical guidance for patients with a history of inadequate glycaemic control but no complications

- Target near-normal HbA_1c_ in this group of patients, but aim for a more gradual reduction in HbA_1c_ compared with newly diagnosed individuals.
- Reassess the potential reasons for inadequate glycaemic control, such as overly conservative management (including delay in introducing combination therapy), inadequate adherence to antidiabetic regimens and inappropriate choice of agents (e.g. agents that do not address the underlying pathophysiology).
- Implement structured educational programmes to motivate individuals with type 2 diabetes to assume a more active role in managing their condition.

### History of CVD

*Definition of patient type*: Known history of CVD, likely to have large pill burden and restrictions on choice of therapy because of comorbidities.

Insight regarding this patient population is provided by individuals who participated in ACCORD, ADVANCE and VADT. These patients had a history of poor glycaemic control, e.g. long duration of diabetes (8–11 years duration at baseline), high baseline HbA_1c_ and a high prevalence of CVD (1,3,4) and these individuals are considered to be at particularly high risk. However, prevention or reduction of complications should be balanced against the need to ensure patient safety. A highly intensive regimen as used in ACCORD, which included rapid dose escalation and introduction of polypharmacotherapy if target HbA_1c_ was not achieved, was associated with a 22% increase in all-cause mortality (1), but preliminary analyses suggest that it was not the individuals with either the fastest or the greatest lowering of blood glucose that incurred this mortality (26). These results suggest that very intensive regimens may be inappropriate for some patients with more advanced diabetes, thus highlighting the need to tailor treatment to the individual. For example, a recent position statement from the American Diabetes Association, American College of Cardiology and American Heart Association on intensive glycaemic control and the prevention of CV events identified particularly high risk individuals as those with a very long duration of diabetes, a known history of severe hypoglycaemia, advanced atherosclerosis or advanced age/frailty (27).

Patients with a history of CVD who do not respond to aggressive glucose-lowering strategies may be more susceptible to CV events, as observed in ACCORD (26) and individualisation of treatment as well as HbA_1c_ targets is needed (1,4). Further detail is provided in ‘Individuals at risk of hypoglycaemia’.

#### Practical guidance for individuals with a history of inadequate glycaemic control and CVD

- Guidance is as for patients with a history of inadequate glycaemic control but no complications, but taking particular care to avoid hypoglycaemia in this group of patients.
- Cardiovascular risk management should be intensified in these individuals.
- It is also important to adopt less stringent glycaemic targets and aim for a more gradual reduction in HbA_1c_.
- Be vigilant to contraindications and other limitations concerning choice of agents, bearing in mind the possibility of drug interactions in this patient group.

#### Practical guidance for individuals at risk of hypoglycaemia

- Educate patients on how to be alert to the possibility of hypoglycaemia, aiming to increase awareness and responsiveness to symptoms of hypoglycaemia.
- Counsel particularly vulnerable patients such as elderly people on the increased risk of hypoglycaemia with irregular lifestyles/eating patterns and encourage compliance to prescribed regimens.
- Emphasise the importance of regular self-monitoring of glucose wherever appropriate.

## Individuals at risk of hypoglycaemia

*Definition of patient type*: Individuals with previous symptoms of hypoglycaemia, those with particularly wide daily glucose fluctuations and individuals such as elderly people who often have impaired creatinine clearance in addition to irregular lifestyles/eating patterns leading to increased susceptibility to hypoglycaemia, especially when taking hypoglycaemic agents such as insulin and sulphonylureas.

Some therapeutic regimens are associated with a significantly increased risk of hypoglycaemia in patients with type 2 diabetes (1,3,4,19). In the UKPDS, the annual rates of major hypoglycaemia were 0.7% with conventional treatment, 1.0% with chlorpropamide, 1.4% with glibenclamide and 1.8% with insulin. This risk was much higher in ACCORD, in which the frequency of severe hypoglycaemia (participants with one or more episodes during the study) was 16.2% with intensive therapy vs. 5.1% with conventional treatment. In ADVANCE, the frequency was 2.7% and 1.5% for intensive and conventional control, respectively, and in VADT it was 21.2% and 9.9% (27). Note the lower incidence in ADVANCE, which may reflect a more gradual reduction in HbA_1c_ over time, although the definition of severe hypoglycaemia did vary considerably between studies. The impact of intensive glycaemic control is also observed in a meta-analysis based on pooled data from UKPDS, PROactive, ADVANCE, VADT and ACCORD, with twice as many people in the intensive control group (2.3%) having a severe hypoglycaemic episode vs. the standard control group (1.2%) (5).

In both ACCORD and VADT, an association was observed between severe hypoglycaemia and CV events, although no cause and effect relationship could be demonstrated (17). Hypoglycaemia may in fact be a marker of other features that may be associated with increased mortality, e.g. non-adherence or autonomic neuropathy, a strong risk factor for sudden death (17).

Other studies also demonstrate the risks associated with hypoglycaemia. For example, in a retrospective analysis, increased risk of mortality was observed in patients with persistent in-hospital hypoglycaemia following admission for acute MI (28).

Hypoglycaemia is an important safety concern, especially in older patients with type 2 diabetes and, in particular, in those with previous CV events (17). Targets should be individualised according to the risk of hypoglycaemia, e.g. history of severe or frequent hypoglycaemia, kidney function, age of patient and previous CV events.

## Conclusions

Good glycaemic control continues to have an essential role in type 2 diabetes management. Our recently updated ‘10 steps to get more type 2 diabetes patients to goal’ (2) remains the blueprint in terms of practical guidance for helping patients to achieve and maintain their glycaemic targets. However, having reviewed the evidence, we recognise that individualising targets and/or treatment according to patient type is paramount. For example, while early intervention is preferred wherever appropriate, certain high risk groups may not respond to overly intensive glucose-lowering regimens such as that utilised in ACCORD.

In this article, we have identified which patient groups require special consideration and provided practical guidance specific to each group. While some of our recommendations apply across all patient groups, others are particularly applicable for certain patients or else require individualisation to achieve the optimal risk:benefit in terms of patient outcomes, as summarised in Table 1. We hope that by providing structured advice on how to tailor treatment according to the individual, we are one step closer in our quest for the best possible outcomes for our type 2 diabetes patients.

## Appendix

*Global Partnership for Effective Diabetes Management* members: George Alberti, University of Newcastle upon Tyne, Newcastle upon Tyne, UK; Pablo Aschner, Javeriana University School of Medicine, Bogota, Colombia; Clifford J Bailey, Aston University, Birmingham, UK; Lawrence Blonde, Ochsner Medical Center, New Orleans, LA, USA; Stefano Del Prato, University of Pisa, Pisa, Italy (Chair); Anne-Marie Felton, Federation of European Nurses in Diabetes, London, UK; Ramon Gomis, Hospital Clinic, Barcelona, Spain; Edward Horton, Joslin Diabetes Center, Boston, MA, USA; James LaSalle, Medical Arts Research Collaborative, Excelsior Springs, MO, USA; Hong-Kyu Lee, Seoul National University, College of Medicine, Seoul, Korea; Lawrence A. Leiter, St Michael’s Hospital and University of Toronto, Toronto, ON, Canada; Stephan Matthaei, Diabetes-Center Quakenbrück, Quakenbrück, Germany; Marg McGill, Diabetes Centre, Royal Prince Alfred Hospital, Sydney, Australia; Neil Munro, Beta Cell Diabetes Centre, Chelsea and Westminster Hospital, London, UK; Richard Nesto, Lahey Clinic, Burlington, MA, USA; Paul Zimmet, Baker IDI Heart and Diabetes Institute, Caulfield, Australia; Bernard Zinman, Samuel Lunefield Research Institute, Mount Sinai Hospital, University of Toronto, Toronto, Canada.
